# Can teletechnology improve patient experience and reduce the use of health care resource?

**Published:** 2012-06-16

**Authors:** David Graeme Smithard, Kim Lee, Sue Williams, Hazel Price, Sharon Lee

**Affiliations:** Kent Community Health, UK; Kent Community Health, UK; Kent Community Health, UK; Kent Community Health, UK; Kent Community Health, UK

**Keywords:** telehealth, long-term conditions, patient experience

## Abstract

**Introduction:**

By 2050 it is estimated that there will be 16 million people over the age of 65 years. With the expansion in the older population and improvements in health care the number people living with a chronic health condition (Long Term Condition, LTC) is increasing. Many people will have more than one LTC e.g. diabetes and cardiac disease. With pressure on the health economy and available resources increasing, managing as many people outside of hospital as appropriately possible is essential. The white paper “Our health our care our say” (DH 2006), challenges local health and social care communities to deliver more care closer to the patients own home. The Kent Telehealth pilot study, undertaken in 2005, investigated whether the use of Telehealth in the UK health care setting could replicate the outcomes of the Veterans Administration programme in the US.

**Methods:**

The pilot examined the role of Telehealth in supporting users and their carers, and assessed its impact on hospital admissions, length of stay, GP contact and nursing visits. Patients acted as their own controls. SF12 and QuIL were used for the qualitative evaluation. Health Ethics approval was granted. All participants provided informed consent. Those meeting the eligibility criteria—of at least one LTC (diabetes, COPD, heart failure) were recruited, equipment was provided to record their vital signs. Vital signs parameters were agreed for individual users with their clinician. Data were automatically uploaded to a web based server, accessible to health care staff responsible for the care of the individual. The frequency of data review was dependent on the service delivery model and appropriate communications were undertaken with the user to facilitate any change in their agreed management plan.

**Results:**

Two hundred and fifty users were recruited, data were available for 202 users for the final analysis. There were 88 less A&E visits and 536 bed days were saved. If admitted the length of stay was shorter by up to 4 days. There was a 28% reduction in calls to the GP, a 23% reduction in visits to the surgery, and an 18% reduction in home visits. It has been estimated that over a six-month period, Telehealth intervention saved an average of £1878 per user (£1038 to £2718, p=0.01). Using Hospital Episode Statistics estimates savings that could be generated across Kent (2006–2007 prices) could be £7.56 million (CI £4.18 million to £10.942 million) annually. Users reported an increased peace of mind, increase quality of life with increased empowerment and self management with improvements in SF12 scores improved for General Health +5.7, for Physical health +8.7.

**Conclusions:**

Telehealth is a potentially valuable adjunct in the management of people with LTCs. Patients become more empowered and independent and as a result, reduced their reliance on primary and secondary care. There is the potential for significant financial gains to be realised, through improved working and reduction in attendance at hospital for admission and or outpatient consultations. Patient quality of life also improved which impacts on how and when they interact with services.

